# 

**Published:** 1877-11-20

**Authors:** 


☞ All communications relating to the business of THE RECORD, for the year 1877, must be addressed to

DR. R. C. WORD,
Business Manager Southern Med. Rec.,
Atlanta, Ga.

☞ Brief and practical communications are solicited on all subjects pertaining to medicine. also reports of cases in practice.

☞ Send money by check, postal order or registered letter.

☞ Write your name, post-office, county and State plainly.

### COLABORATORS AND CONTRIBUTORS.

L. B. Anderson, M.D., Va.
Swan M. Burnett, M.D., D.'C
J. M. Bristow M.D., Texas
J. W. Booth, M.D., N C.
W. F. Barr, M.D., Va.
B. W. Corn, M.D., Texas
John Castor, M.D., Iowa.
E. T. Easley. A.M., M.D., Ark
A. H. Goelet, M D., N. Y.
E. C. Gehrung, M.D., Mo.
G. D. Hodge, M.D., Ark.
S. M. Hogan, M.D., Ala.
A. McLane Hamilton, M.D.,NY
C. C. Vander[illegible]eck, M.D., Pa.
Z. B. Herndon, M.D., Va.
Lindsay Johnson, M.D., Ga.
A. R. Kilpatrick, M.D., Tex.
R. Kells, M.D., Miss.
W. L. Lipscomb, M.D., Miss.
J. M. Lewis, M.D., Miss.
G. A. Dyer, M.D., Ind.
C H Lathrop, M.D., Iowa.
A A Lyon, M.D., La
M Lewis, M.D., Tenn
G M Rivers, M.D , S C
A S ayne, M.D., Va.
A. M. Whitehead, M.D., O.

